# Molecular Research on Drug Induced Liver Injury

**DOI:** 10.3390/ijms19010216

**Published:** 2018-01-11

**Authors:** Rolf Teschke, Gaby Danan

**Affiliations:** 1 Department of Internal Medicine II, Division of Gastroenterology and Hepatology, Klinikum Hanau, Academic Teaching Hospital of the Medical Faculty, Goethe University Frankfurt/Main, Frankfurt am Main, D-63450 Hanau, Germany; 2 Pharmacovigilance Consultancy, 75020 Paris, France

Drugs may cause liver injury in a few susceptible individuals, but the molecular events that lead to this idiosyncratic, largely dose-independent and non-predictable drug-induced liver injury (DILI) are mostly unknown, since animal models to explore the pathogenetic mechanisms of human idiosyncratic DILI are not yet reliable. More is known about molecular events in connection with the intrinsic dose-dependent DILI, since animal studies can clarify some questions of the underlying pathogenetic conditions. Molecular risk factors of idiosyncratic DILI include drug lipophilicity, high daily dose, high metabolic rate, and drug–host interactions, in addition to genetic factors that may initiate liver injury. Nevertheless, many pathogenetic challenges at the molecular level remain unknown. Uncertainties also relate to possible molecular diagnostic biomarkers, and whether these could assist RUCAM (Roussel Uclaf Causality Assessing Method) in establishing causality for suspected drugs in DILI cases. Clearly, much more research is still warranted to elucidate molecular pathogenetic events in DILI which may help to establish diagnostic biomarkers. The aim of this Special Issue is to provide a broad updated overview on these molecular related hepatotoxicity features with their challenges and highlights and to encourage more molecular research in DILI. Experts in the field contributed their views to this emerging and fascinating topic of molecular aspects. Since some topics are still controversial, we expect and appreciate lively discussions.

Hallmarks of clinical liver injury will be presented in the Special Issue “Molecular Research on Drug Induced Liver Injury” published in the *International Journal of Molecular Sciences* to address the progress and current standing in the vast field of liver injury. A total of 10 reviews and original articles [1,2,3,4,5,6,7,8,9,10] have been published in this Special Issue, as detailed in Table 1. 

The Special Issue opens with the review article of Iorga et al. [1], who explain some of the pathogenetic events leading to cell death, apoptosis, and necrosis in intrinsic and idiosyncratic drug-induced liver injury (DILI). While intrinsic DILI is caused, for instance, by acetaminophen and is triggered by oxidative stress of the endoplasmic reticulum or mitochondria, idiosyncratic DILI is the prevailing injury type for most drugs and is commonly linked to the immune system. Due to the variability of potentially hepatotoxic drugs and the diversity of the underlying pathogenetic background, establishing a unique biomarker for all drugs and both DILI forms is hardly achievable. Unquestionably, biomarkers can only be validly established if results of the studies are based on liver injury cases with high causality gradings of the suspected drug, preferentially using the worldwide most commonly applied RUCAM (Roussel Uclaf Causality Assessment Method). 

In the second article, Teschke et al. [2] expand the topic of diagnostic biomarkers in DILI and discuss advantages and shortcomings of biomarkers that are presently under investigation. Biomarkers are single proteins or a panel of proteins, nucleic acids, or protein–metabolite adducts. However, most published biomarkers of idiosyncratic DILI merely assess liver injury and lack identification of the suspected drug. Some serum biomarkers are in clinical usage for intrinsic DILI by acetaminophen (acetaminophen–protein adducts), Germander (microsomal epoxide hydrolase), or San Chi (pyrrole–protein adducts). Since no valid biomarkers are available for idiosyncratic DILI, its diagnosis rests on the RUCAM, which provides reliable quantitative causality gradings and transparent case data with individual RUCAM scores. These transparent RUCAM data are easily reassessable by peers, as opposed to cases evaluated by intransparent global introspection. 

Subsequently, McEuen et al. [3] consider risk factors of DILI such as drug lipophilicity and metabolism. As a result of their studies, the combination of high daily dose (≥100 mg) and lipophilicity was associated with an increased risk of DILI, as was the combination of extensive hepatic metabolism (≥50%) and high daily dose (≥100 mg). Although their data were convincingly presented, uncertainty remains on the quality of DILI cases that were used for their studies. Cases of most DILI studies are confronted with various confounders that put the diagnosis of DILI in question and do not allow valid interpretation of results. With respect to the present report, confounders include how alternative causes were excluded and how cases with missing data were handled. Finally, the authors correctly mentioned that their data needs to be evaluated by accepted methods for causality assessment such as RUCAM, certainly a good proposal for data verification.

In the next clinically oriented review article, Sebode et al. [4] present new insights into the two different diseases—genuine autoimmune hepatitis (AIH) and the autoimmune-like idiosyncratic drug- and herb-induced liver injury—which can mimic characteristics of AIH. These include clinical features, appearance of serum autoantibodies, and infiltration of the liver by immune-competent cells, impeding clinical differentiation. The authors argue that the diagnosis of DILI is difficult to make but is supported by scores of causality assessment such as RUCAM, which provides the exclusion of acute viral hepatitis, AIH, and other liver diseases. The use of an immunosuppressive therapy for the autoimmune form of DILI is controversially discussed and based on little evidence, so recommendations for therapy are limited to stopping the causative agent. 

Based on studies in humans and animals, Kim et al. [5] explored the metabolic characteristics of ketoconazole (KCZ), known as an antifungal drug and for its potential hepatotoxicity. A total of 28 metabolites were found, and 11 were novel and identified in this study. Newly identified metabolites in human were comparable to those in mice, and were classified into three categories according to the metabolic positions of a piperazine ring, imidazole ring, and *N*-acetyl moiety. Three cyanide adducts of KCZ were identified in mouse and human microsomal incubates as “flags” to trigger additional toxicity study. The oxidation of piperazine to iminium is likely the bioactivating step of toxicity. Therefore, this metabolomics approach contributes to our understanding of KCZ liver injury and asks to what extent these findings are transferable to clinical conditions of liver injury in patients under antifungal therapy with KCZ. 

Another clinical issue supported by experimental toxicity studies was addressed by Goda et al. [6]. They make the point that during drug development, fatty changes of the hepatocytes are sometimes detected in animals, caused by mitochondrial dysfunction triggered by the drug under consideration. Fatty changes reflect triglycerides containing long-chain fatty acids, which are the main energy source of the mitochondria. Accumulation of these triglycerides is considered to be related to the inhibition of the mitochondrial respiratory chain, as evidenced by in vitro mitochondria toxicity studies. The conclusion was reached that fatty changes of the hepatocytes in pre-clinical studies of drug candidates can be regarded as a critical finding for the estimation of their potential risk to cause DILI in humans, provided the fatty change is due to mitochondrial dysfunction of the respiratory chain.

Turning now to the next clinical topic supported by in vitro studies, Hirasawa et al. [7] focused on pathogenetic aspects of liver injury caused by ximelagatran, previously used as a thrombin inhibitor but soon withdrawn from the market. The authors reported on a series of in silico simulations and in vitro measurements of ximelagatran bound to human leukocyte antigen (HLA)- DR (D Related) molecules. They presented data of docking studies whereby ximelagatran has a higher potential to interact with the ligand peptide of HLA molecules-binding groove than its active metabolite melagatran. Based on this direct interaction, ximelagatran competes with the ligand peptide for the binding site. This competition is associated with a disruption of the binding of the respective peptides at the binding site. This in turn could alter the antigen loading and cause the immune response, which finally leads to the idiosyncratic ximelagatran-induced hepatotoxicity.

Using two different experimental liver injury models in mice, Yamamotoya et al. [8] studied the role of LUBAC (linear ubiquitin chain assembly complex), composed of SHARPIN (SHANK-associated RH domain-interacting protein) and other proteins, forms linear ubiquitin on nuclear factor-κB (NF-κB) essential modulator (NEMO) and induces NF-κB pathway activation in acute liver injury and liver cirrhosis induced by carbon tetrachloride or acetaminophen. SHARPIN expression was suppressed and LUBAC formation was significantly reduced in the livers of mice 24 h after injection of either carbon tetrachloride or acetaminophen. Other experiments provided evidence that LUBAC is essential for the survival of hepatocytes and its reduction promotes hepatocyte death in addition of the direct effect of drug toxicity. Additional suggestions have been made that SHARPIN knockdown by adenoviral transfer of the corresponding RNA into the mouse livers rapidly caused massive hepatocyte death together with inflammation and fibrosis. It remains to be established how these experimental results can be transferred to clinical hepatology. 

Experimental hepatotoxicity due to the anti-tuberculosis drug rifampicin was induced in mice, which led Kim et al. [9] to investigate the mechanism of this special liver injury. Based on the biochemical parameters in the plasma after rifampicin treatment, the hepatotoxic effect of rifampicin in the mouse liver was defined as a mixed liver injury. Using comparative global proteomics analyses, they identified 1101 proteins in the liver and quantified 1038 proteins. Some of the proteins were up-regulated and others were down-regulated, and the extent of the changes was dependent on the dose of rifampicin given (which was 177 and 442.5 mg/kg rifampicin). More specifically, glutathione transferase activity was up-regulated and proteins related to arachidonic acid metabolism were down-regulated. The clinical relevance of these results remains to be elucidated, especially since rifampicin alone is exceptionally hepatotoxic in humans, and it is rather the combination with isoniazid and/or pyrazinamide and is known as a strong CYP inducer.

In another experimental study carried out in rats, Xiang et al. [10] reported on the effects of a novel oleanolic acid derivative Oxy-Di-AO on CCl_4_-induced hepatic fibrosis. As a derivative of the hepatoprotective oleanolic acid originally from *Ligustrum lucidum*, the synthetic Oxy-Di-AO has a protective effect and prevented the development of CCl_4_-induced liver fibrosis when given intraperitoneally daily for 9 weeks. The protective effect was verified by measurements of ALT (alanine transaminase) and AST (aspartate transaminase) and by histological and histochemical analyses. After single-dose oral administration, pharmacokinetic studies showed that time to reach peak concentration of Oxy-Di-AO was 12 h, associated with an elimination half-life of 2.19 h. The LD_50_ value of Oxy-Di-OA exceeded 2000 mg/kg via intraperitoneal injection in mice. Whether Oxy-Di-AO is useful in clinical hepatology remains to be established.

As guest editors of this Special Issue with overall 10 contributions, we thank all experts who devoted their time and energy to writing their papers. For each submitted paper, the editorial team chose at least three experts in the field as external and independent reviewers whose recommendations were highly appreciated and substantially improved the quality of the articles as they were finally accepted for publication. These publications covered the broad field of molecular aspects of liver injury by drugs and also illustrate how crucial it is to assess pathogenetic events. It was a pleasure for us to work together with the authors and such a competent editorial team to get this exciting job done. 

Thank you again to all who contributed and assisted to get this Special Issue published.

## Figures and Tables

**Table 1:** Summary of papers in the Special Issue.

Authors	Title	Topics/Keywords	Type
Iorga et al. [1]	Drug-Induced Liver Injury: Cascade of Events Leading to Cell Death, Apoptosis or Necrosis	hepatotoxicity; human leukocyte antigen (HLA); adaptation; acetaminophen; DILI; apoptosis; necrosis	Review
Teschke et al. [2]	Drug Induced Liver Injury: Can Biomarkers Assist RUCAM in Causality Assessment?	drug induced liver injury; DILI; RUCAM; Roussel Uclaf Causality Assessment Method; diagnostic biomarkers; pathogenesis	Review
McEuen et al. [3]	Associations of Drug Lipophilicity and Extent of Metabolism with Drug-Induced Liver Injury	hepatotoxicity; drug dose; drug lipophilicity; metabolism; risk factor; annotation	Review
Sebode et al. [4]	“Autoimmune(-Like)” Drug and Herb Induced Liver Injury: New Insights into Molecular Pathogenesis	idiosyncratic; drug-induced liver injury; autoimmune hepatitis; herbal and dietary supplements; herbs; autoimmune-like drug induced liver injury	Review
Kim et al. [5]	Revisiting the Metabolism and Bioactivation of Ketoconazole in Human and Mouse Using Liquid Chromatography–Mass Spectrometry-Based Metabolomics	ketoconazole; metabolite profiling; bioactivation; metabolomics	Article
Goda et al. [6]	Evaluation of the Potential Risk of Drugs to Induce Hepatotoxicity in Human—Relationships between Hepatic Steatosis Observed in Non-Clinical Toxicity Study and Hepatotoxicity in Humans	steatosis; drug-induced liver injury; lipidomics; mitochondrial dysfunction	Article
Hirasawa et al. [7]	In Silico and in Vitro Analysis of Interaction between Ximelagatran and Human Leukocyte Antigen (HLA)-DRB1*07:01	ximelagatran; melagatran; HLA (human leukocyte antigen); hepatotoxicity; IDT (idiosyncratic drug toxicity); MD (molecular dynamics) simulation	Article
Yamamotoya et al. [8]	Reduced SHARPIN and LUBAC Formation May Contribute to CCl_4_- or Acetaminophen-Induced Liver Cirrhosis in Mice	LUBAC; CCl_4_ and APAP-induced hepatitis; inflammation; fibrosis; hepatocyte apoptosis	Article
Kim et al. [9]	Mechanism Investigation of Rifampicin-Induced Liver Injury Using Comparative Toxicoproteomics in Mice	rifampicin; drug-induced liver injury; anti-tuberculosis; proteomics	Article
Xiang et al. [10]	A New Oleanolic Acid Derivative against CCl_4_-Induced Hepatic Fibrosis in Rats	oleanolic acid derivative; carbon tetrachloride-induced; liver fibrosis; histological study; acute toxic test; pharmacokinetic	Article
